# Kinetic analysis of [^11^C]befloxatone in the human brain, a selective radioligand to image monoamine oxidase A

**DOI:** 10.1186/2191-219X-3-78

**Published:** 2013-11-25

**Authors:** Paolo Zanotti-Fregonara, Claire Leroy, Dimitri Roumenov, Christian Trichard, Jean-Luc Martinot, Michel Bottlaender

**Affiliations:** 1 University of Bordeaux, CNRS, INCIA, UMR 5287, Talence 33400, France; 2 CEA, DSV, I2BM, Service Hospitalier Frederic Joliot, Orsay 91401, France; 3 INSERM, CEA, ParisSud University, U.1000, Orsay 91400, France; 4 CEA, DSV, I2BM, Neurospin, Gif-sur-Yvette 91191, France

**Keywords:** [^11^C]Befloxatone, Monoamine oxidase A, PET, Spectral analysis

## Abstract

**Background:**

[^11^C]Befloxatone measures the density of the enzyme monoamine oxidase A (MAO-A) in the brain. MAO-A is responsible for the degradation of different neurotransmitters and is implicated in several neurologic and psychiatric illnesses. This study sought to estimate the distribution volume (*V*_T_) values of [^11^C]befloxatone in humans using an arterial input function.

**Methods:**

Seven healthy volunteers were imaged with positron emission tomography (PET) after [^11^C]befloxatone injection. Kinetic analysis was performed using an arterial input function in association with compartmental modeling and with the Logan plot, multilinear analysis (MA1), and standard spectral analysis (SA) at both the regional and voxel level. Arterialized venous samples were drawn as an alternative and less invasive input function.

**Results:**

An unconstrained two-compartment model reliably quantified *V*_T_ values in large brain regions. A constrained model did not significantly improve *V*_T_ identifiability. Similar *V*_T_ results were obtained using SA; however, the Logan plot and MA1 slightly underestimated *V*_T_ values (about -10%). At the voxel level, SA showed a very small bias (+2%) compared to compartmental modeling, Logan severely underestimated *V*_T_ values, and voxel-wise images obtained with MA1 were too noisy to be reliably quantified. Arterialized venous blood samples did not provide a satisfactory alternative input function as the Logan-*V*_T_ regional values were not comparable to those obtained with arterial sampling in all subjects.

**Conclusions:**

Binding of [^11^C]befloxatone to MAO-A can be quantified using an arterial input function and a two-compartment model or, in parametric images, with SA.

## Background

Monoamine oxidase (MAO) is a mitochondrial enzyme responsible for the degradation of several neurotransmitters, such as dopamine, serotonin, and noradrenaline. Two MAO isoforms - products of different genes - have been identified in mammals: enzyme monoamine oxidase A (MAO-A), which is primarily found in cathecolaminergic neurons, and MAO-B, which is mainly present in glial cells and serotonergic neurons [1]. MAO-A is widespread in the human brain; the highest concentrations are found in the thalamus and the occipital cortex, intermediate values are found in the basal ganglia, and lower concentrations are seen in the frontal cortex and cerebellum [1,2]. MAO-A dysfunction has been implicated in several neurologic illnesses such as Parkinson's disease, Alzheimer's disease, and Huntington's disease, as well as in psychiatric diseases such as major depressive disorder [3]. Notably, several studies have linked MAO-A enzyme activity and gene expression to tobacco addiction [2,4-7].

With some exceptions [6], studies on MAO-A activity have been hindered by the lack of suitable ligands for this enzyme. For instance, the metabolism rate of [^11^C]harmine in the plasma is very high, thus making a reliable quantification of the input function challenging [8], and [^11^C]brofaromine has very little displaceable binding [9].

Befloxatone is a potent, selective, and reversible inhibitor of brain MAO-A activity [10]. It can be radiolabeled with ^11^C using [^11^C]phosgene as the reagent [11]. [^11^C]Befloxatone has a highly specific binding to MAO-A, as shown by extensive *in vitro*[10] and *in vivo* studies in primates [12]. In particular, nonsaturable uptake, obtained after a pretreatment with a high dose of unlabeled befloxatone, is very low and represents only 3% of the total uptake [12]. [^11^C]Befloxatone has good imaging characteristics and has been used in positron emission tomography (PET) studies to quantify *in vivo* MAO-A in rats [13], nonhuman primates [12,14], and humans. In humans, it has been used to compare MAO-A binding potential in smokers and nonsmokers [7]; however, a rigorous kinetic modeling study in humans has never been performed. This paper sought to (1) quantify the uptake of [^11^C]befloxatone in the human brain, both at the regional and voxel level, using an arterial input function; and (2) explore the possibility of replacing the arterial input function with a less invasive approach based on sampling of arterialized venous blood.

## Methods

### Subjects

Seven healthy, nonsmoker, male volunteers (72 ± 9.5 kg, age 26 ± 4.5 years) from a previous clinical protocol were included in this study [7]. All subjects were free of current medical or psychiatric illnesses and had no history of drug or alcohol abuse. Subjects were instructed to avoid monoamine-rich food 3 days before and alcohol consumption 1 week before imaging. Vital signs (blood pressure, heart rate) were recorded before [^11^C]befloxatone injection and at the end of the scan. The study was approved by the regional ethics committee for biomedical research at the Bicêtre Hospital. Each subject gave a written informed consent.

### Brain image acquisition

PET scans were performed on an ECAT EXACT HR + scanner (Siemens Medical Solutions, Knoxville, TN, USA). One transmission scan was acquired before intravenous bolus injection of [^11^C]befloxatone (290 ± 24.4 MBq). The specific activity was 35.2 ± 14.9 GBq/μmol, which is within the range of the values usually obtained from the synthesis of [^11^C]befloxatone [11] and sufficient to rule out any mass effect. Even when considering a conservative *B*_max_ value of 99 pmol/mL (as found *in vitro* in the human cerebellum [14]), the *B*_max_ occupancy is well below 1% in each subject. Dynamic three-dimensional images were acquired over 60 min in 29 frames, whose duration ranged from 10 s to 5 min. Images were reconstructed with filtered back projection.

Each subject also underwent brain T1-weighed three-dimensional magnetic resonance imaging (MRI) using a 1.5 T Signa scanner (GE Healthcare, Milwaukee, WI, USA). MRI images were acquired with a voxel size of 0.938 × 0.938 × 1.3 mm^3^ and a thickness of 1.3 mm in axial slices.

### Measurement of [^11^C]befloxatone in arterial and venous plasma

Arterial blood samples were drawn from the radial artery of each subject in 10-s intervals until 2 min, followed by samples at 3, 4, and 5 min and then every 5 min until the end of the scan at 60 min. About 27 samples were drawn for each subject. For each blood sample, the decay-corrected concentration of [^11^C]befloxatone was measured in whole blood and plasma. Plasma was obtained by centrifugation. Plasma-free fraction was not measured. Venous blood samples were simultaneously drawn from the cubital vein of the opposite arm, using the same number of samples and time schedule as the arterial ones. As for arterial blood, plasma was separated from total venous blood. Arterialization of venous blood was achieved by heating the arm with hot water bags, starting 30 min before ligand injection until the end of the scan. The bags were replaced every 20 to 30 min. Four or five times during each scan, blood gases (pO_2_ and pCO_2_) were measured in five subjects.

The whole plasma time-activity curves were taken as input functions because in a previous series of (yet unpublished) four human scans from our laboratory, chromatograms obtained with high-performance liquid chromatography (HPLC) did not show any detectable radiometabolites in the plasma, even at late time points. The technique we used to measure the amount of unchanged radiotracer in the plasma was similar to that described in [14]. Specifically, after deproteinization with acetonitrile, the samples were centrifuged and the supernatant was injected directly into the HPLC column. A reverse-phase μBondapak C18 column (300 × 7.8 mm, 10 μm; Waters, Milford, MA, USA) was eluted applying a gradient from 20% acetonitrile in 0.01 M phosphoric acid up to 80% in 5.5 min, to 90% at 7.5 min, and returning to 20% at 7.6 min with a total run length of 10 min. The flow rate of the eluent was maintained at 6 mL/min. Befloxatone was eluted with a retention time of 6 min. Data acquisition and analysis were carried out using Winflow software (version 1.21, JMBS Developments, Grenoble, France).

### Brain image analysis

The summed PET image, obtained by averaging all frames, was first coregistered to the individual MRI using SPM5 (Wellcome Department of Cognitive Neurology, London, UK). MRI and PET images were then normalized to the Montreal Neurologic Institute (MNI) space using the transformation parameters from the MRI images. A template of regions of interest (ROIs) [15] was used to extract brain time-activity curves for the following 13 regions: anterior and posterior cingulate cortices, caudate, putamen, thalamus, pons, cerebellum, hippocampus, parahippocampus, and the prefrontal, parietal, temporal, and occipital cortices. Each region was obtained from the weighted average of the left and right region. Image analysis and kinetic modeling were performed with Pmod 3.1 (Pmod Technologies, Zurich, Switzerland).

### Calculation of distribution volume

Because [^11^C]befloxatone is a reversible MAO-A inhibitor [11], kinetic analysis was performed using one- (1TCM) and two-tissue compartment (2TCM) model to calculate the total distribution volume (*V*_T_), which equals the ratio at equilibrium of the concentration of radioligand in the brain to that in the plasma [16]. *V*_T_ is the sum of the binding in the specific compartment (*V*_S_) and in the nondisplaceable compartment (*V*_ND_). In theory, *V*_T_ may change not only as a function of *V*_S_ but also of *V*_ND_. However, in kinetic modeling studies, *V*_ND_ is commonly assumed to be constant across different brain regions and across different subjects of the same species. Thus, variations of *V*_T_ are considered to reflect variations of *V*_S_ values. For [^11^C]befloxatone in particular, *in vivo* studies in primates showed that, after pharmacological blockade, the residual radioactive concentrations were very low and identical in all brain structures [11]. The 2TCM analysis was performed in two ways, (1) without constraining the rate constants and (2) fixing the ratio *K*_1_/*k*_2_ to the value obtained in the whole brain, to improve the identifiability of parameters. The individual rate constants were estimated with the weighted least square method and with the Marquardt optimizer. Brain activity was corrected for its vascular component using the measured whole blood concentrations, assuming that the cerebral blood volume is 5% of the total brain volume [17]. The delay of radiotracer arrival between the radial artery and the brain was corrected by fitting the whole brain. The plasma input function was modeled using a linear interpolation of the [^11^C]befloxatone concentrations before the peak and a tri-exponential fit of concentrations after the peak. Kinetic modeling was also performed at the regional level using the graphical Logan plot (Logan_voi_), Ichise's multilinear analysis (MA1_voi_), and a standard spectral analysis (SA_voi_).

#### Logan plot

The Logan plot is a model-independent graphical method for reversible tracers [18]. It performs a linearization of the data so that, after a certain time (t > t*), the slope can be related to the *V*_T_, according to

(1) $$\frac{\int_{0}^{t}C_{T}\left(\tau \right)\mathrm{d\tau}}{C_{T}\left(t\right)}=V_{T}\frac{\int_{0}^{t}C_{P}\left(\tau \right)\mathrm{d\tau}}{C_{T}\left(t\right)}+c$$

where *C*_T_ represents the tissue concentration and *C*_p_ the plasma activity.

The Logan plot is a computationally quick and robust technique. It may, however, underestimate *V*_T_, especially in the case of small and noisy regions [19].

#### Multilinear analysis

The multilinear analysis (MA1) is a modification of the Logan plot developed to remove noisy measurements (*C*_T_) from the independent variables [20], according to the following equation:

(2) $$C_{T}\left(t\right)=-\frac{V}{b}\int_{0}^{t}C_{p}\left(\tau \right)\mathrm{d\tau}+\frac{1}{b}\int_{0}^{t}C_{T}\left(\tau \right)\mathrm{d\tau}$$

where *V* is the total distribution volume and *b* is the intercept of the Logan plot that becomes constant at t > t*. This approach has been shown to minimize *V*_T_ estimation bias in the case of noisy measurements [20].

#### Spectral analysis

This technique is based on a time-invariant single input/single output model used to identify tissue kinetic components [21]. As with the Logan plot, SA does not require prior knowledge of the number of compartments in the system. The tissue concentration, *C*_T_(*t*), is obtained by convolving the plasma time-activity curve, *C*_P_(*t*), with the sum of *M + 1* exponentials, as follows:

(3) $$C_{T}\left(t\right)=\sum_{j=0}^{M}Cp\left(t\right)⊗\alpha_{j}e^{-\beta_{j}t}=\sum_{j=0}^{M}\alpha_{j}\int_{0}^{t}Cp\left(\tau \right)e^{-\beta_{j}\left(t-\tau \right)}\mathrm{d\tau}$$

where α_*j*_ and *β*_*j*_ are assumed to be real-valued and nonnegative.

*V*_T_ was calculated from the estimated spectrum as

(4) $$V_{T}=\sum_{j=1}^{M}\frac{\alpha_{j}}{\beta_{j}}$$

In the present study, the *β*_*j*_ grid was defined with a maximum value of 100 and a logarithmic distribution of *β*_*j*_, *j =* 1, 2… *M*, using a spectral range from 0.01 to 1. SA was performed using the SAKE software [22].

Finally, we sought to determine whether *V*_T_ values could be obtained from voxel-wise analysis. We created parametric images using the Logan plot (Logan_voxel_), multilinear analysis (MA1_voxel_), and standard spectral analysis (SA_voxel_). For Logan_voxel_ and MA1_voxel_, the PET frames used for regression were selected on the basis of the start time obtained from the time-activity curve of the whole brain. For all parametric images, regional values were obtained by averaging the voxel values within each region.

### Statistical analysis

Goodness of fit by nonlinear least squares analysis was evaluated using the Akaike information criterion (AIC) and model selection criterion (MSC). The most appropriate model is that with the smallest AIC and the largest MSC score. Goodness of fit by the one- and two-compartment models was compared with *F* statistics [23]. A value of *P* < 0.05 was considered significant. The identifiability of kinetic variables was expressed as a percentage of the rate constant and calculated as standard error obtained from the diagonal of the covariance matrix [24]. Standard error for *V*_T_ was calculated from the covariance matrix using the generalized form of error propagation equation [25], where correlations among parameters are taken into account. Smaller values indicate better identifiability.

For each subject, regional *V*_T_ values obtained with 2TCM at the regional level were considered as the gold standard. Comparisons to the *V*_T_ calculated with Logan_voi_, MA1_voi_, SA_voi_, Logan_voxel_, MA1_voxel_, and SA_voxel_ were performed with a repeated measures analysis of variance (ANOVA) for *V*_T_ values in the various regions. Statistical analyses were performed with SPSS (version 17 for Windows; SPSS Inc., Chicago, IL, USA).

## Results

### Measurement of [^11^C]befloxatone in arterial and venous plasma

[^11^C]Befloxatone concentrations in arterial plasma peaked about 30 s after injection at 21 ± 7.1 standard uptake value (SUV) and then rapidly decreased. Both the plasma and whole blood arterial curves were well fitted by a tri-exponential function. Venous concentrations peaked about 50 s after injection with an SUV of 6.7 ± 2.6.

**Figure 1:**
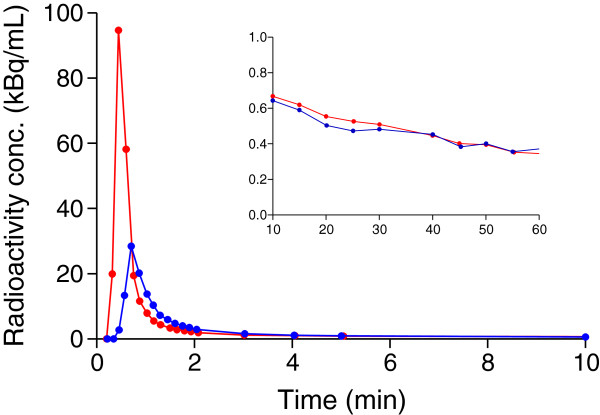
**Plasma time-activity curve of [**^**11**^**C]befloxatone concentrations in arterial and venous blood for a representative subject.** The main frame shows the first 10 min; the inset magnifies the remaining part of the input function. Red curve, arterial blood; blue curve, venous blood. The venous peak was predictably much lower than the arterial one, but [^11^C]befloxatone concentrations were remarkably similar immediately after the peak until the end of the scan. Although compartmental modeling cannot be used with a venous input due to the different shapes of the early part of the curves, the Logan graphical analysis only depends on the total area under the curve. For this subject, the *V*_T_ error using only the venous input was 7.2% compared to the reference *V*_T_ values obtained with the arterial input.

**Figure 2:**
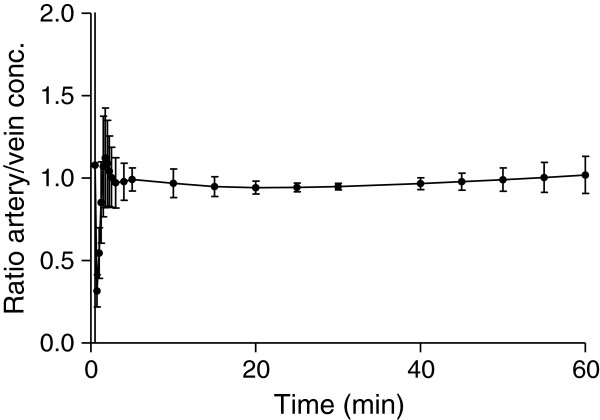
**Arteriovenous difference of radioligand concentrations (mean ± SD) across the different subjects.** Equilibrium was reached within 3 min of injection and remained stable until the end of the scan.

Pairs of arterial and venous samples were sometimes drawn with a few seconds of delay in either direction. Therefore, in order to compare arteriovenous concentrations of the radioligand, we fitted the plasma time-activity curves with a tri-exponential function and interpolated the values at the same grid of standard times. The average pO_2_ measured in venous blood was 77% ± 8% of the pO_2_ measured in the arterial blood. The average pCO_2_ in the vein was 101% ± 1% compared to the arterial value. A stable arteriovenous equilibrium of [^11^C]befloxatone concentrations was found only a few minutes after injection (Figure 1). Average arteriovenous differences were already at about 5% or less within 2.5 min after injection and remained unchanged until the end of the scan (Figure 2). After the peak, in each individual subject the arteriovenous difference was almost always less than 10% at each time point. One subject, however, had differences between 10% and 20% at each time point from 40 min after injection to the end of the scan. A good correlation was found between arterial and venous concentrations at each time point from 3 to 60 min (*n* = 13 pairs). *R*^*2*^ ranged from 0.64 at 3 min to 0.99 at 30 min.

### Brain radioactivity and kinetic analysis

**Figure 3:**
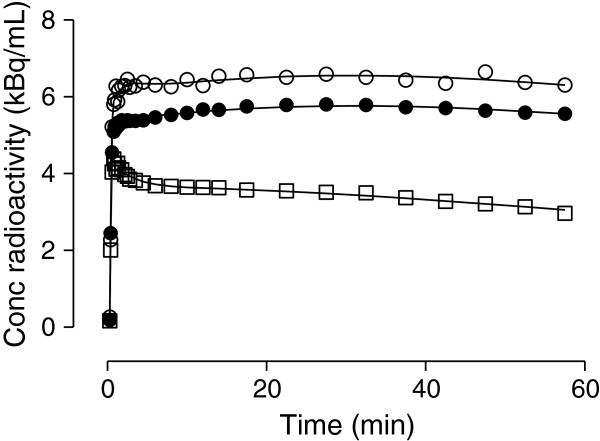
**Representative brain time-activity curves in one subject.** Three regions are shown: putamen (empty circle), parietal cortex (filled circle), and cerebellum (empty square). Lines represent fitting with unconstrained 2TCM.

Radioactivity showed a rapid uptake in the brain, progressively increased during the first 25 min (mean peak value, 1.5 to 2 SUV), and then slowly decreased (Figure 3). These time-activity curves were very similar to those described for [^11^C]befloxatone in nonhuman primates [12].

**Figure 4:**
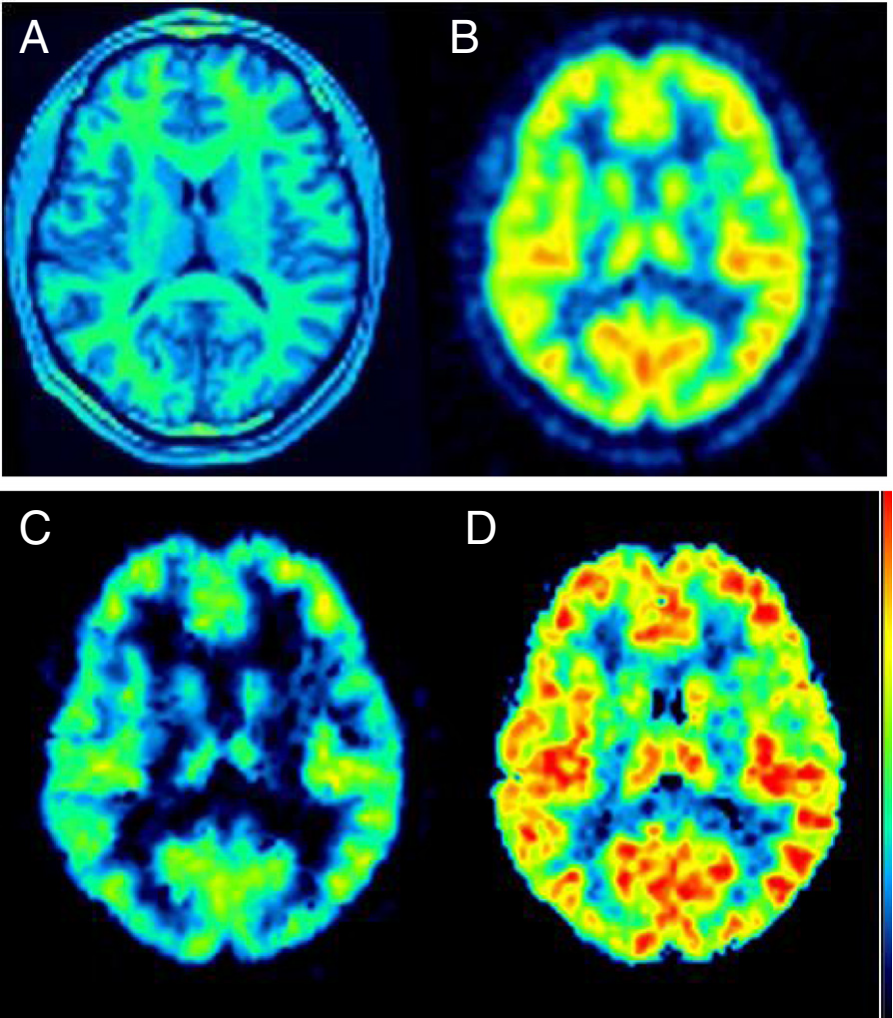
**Brain images.** MRI **(A)**, summed PET **(B)**, and *V*_T_ parametric images in a representative subject. The parametric images were calculated with the Logan plot **(C)** and spectral analysis **(D)** and are shown with the same color scale. Parametric images obtained with MA1 (not shown) were too noisy to be quantified.

Radioactivity uptake was widespread and distributed fairly homogeneously across the brain (Figure 4). Consistent with the known distribution of MAO-A [2], a lower uptake was noted in the cerebellum of all subjects (the cerebellar area under the curve was about 35% lower than the mean value of the whole brain). However, because low amounts of MAO-A are expressed in the cerebellum [26], analysis with a reference region was not used.

The brain kinetic analysis had two major results: (1) [^11^C]befloxatone binding in the brain was better described by a two-tissue compartment model but the 2TCM with a constraint of *K*_1_/*k*_2_ did not provide a better goodness of fit for *V*_T_, and (2) both at the regional and voxel level, SA provided better *V*_T_ estimates than Logan and MA1.

As regards the first finding, unconstrained one- and two-compartment fitting converged in all regions and in all scans. Fitting was visually quite similar using either model; however, the two-tissue compartment model showed lower mean AIC (-22.3) and higher mean MSC (4.2) scores than the one-compartment model (AIC = 1.1, MSC = 3.4). The superiority of 2TCM was confirmed by *F* statistics. In addition, SA showed at least two equilibrating components in all regions for all subjects. The average identifiability across brain regions for two-compartment fitting was 4.7%. The average *V*_T_ value across all brain regions was 14.3 ± 3.0 mL/cm^3^. The highest binding was found in the thalamus (average *V*_T_ of 19.3 ± 1.4 mL/cm^3^), and the lowest in the cerebellum (6.7 ± 0.9 mL/cm^3^) (Table 1). The constrained two-compartment model fitting converged in all regions and in all scans but provided a similar goodness of fit (AIC = -20.8 and MSC = 4.1) as the unconstrained model. The average identifiability across brain regions was 4.4% (versus 4.7% for the unconstrained fitting).

**Table 1:** Distribution volume calculated from compartmental modeling, Logan plot, MA1, and SA using regions or individual voxels

**Region**	***V***_**T **_**(mL/cm**^**3**^**)**					
	**Region**				**Voxel**	
	**Unconstrained two-tissue compartment model**	**Logan**	**MA1**	**SA**	**Logan**	**SA**
Frontal cortex	13.3 ± 0.84 (2.7)	12.5 ± 0.79	11.8 ± 0.71	13.6 ± 1.21	8.66 ± 2.14	13.7 ± 0.90
Temporal cortex	15.1 ± 1.04 (2.6)	14.1 ± 0.90	13.5 ± 0.83	15.3 ± 0.85	9.69 ± 2.64	15.4 ± 0.97
Hippocampus	16.2 ± 1.11 (6.9)	14.8 ± 1.09	13.8 ± 0.75	15.2 ± 1.74	6.36 ± 1.87	14.4 ± 1.79
Parietal cortex	13.0 ± 0.61 (2.8)	12.3 ± 0.53	11.6 ± 0.40	13.3 ± 0.94	8.59 ± 2.20	13.5 ± 1.10
Occipital cortex	15.4 ± 1.08 (2.8)	14.4 ± 0.99	13.6 ± 0.83	15.5 ± 1.20	9.87 ± 2.19	15.7 ± 1.13
Anterior cingulum	16.2 ± 1.56 (4.6)	15.2 ± 1.43	14.7 ± 1.20	16.9 ± 2.02	9.94 ± 2.56	16.2 ± 0.90
Caudate	12.5 ± 1.20 (7.7)	11.4 ± 1.00	11.0 ± 0.73	12.4 ± 1.21	7.17 ± 1.94	13.0 ± 1.36
Putamen	14.8 ± 0.67 (3.8)	13.9 ± 0.77	13.3 ± 0.70	15.2 ± 0.80	10.3 ± 3.00	16.5 ± 0.99
Thalamus	19.3 ± 1.43 (5.6)	17.7 ± 1.69	16.5 ± 1.18	19.5 ± 2.33	10.1 ± 3.11	19.0 ± 2.32
Cerebellum	6.73 ± 0.91 (2.5)	6.42 ± 0.82	6.18 ± 0.75	6.83 ± 0.95	5.19 ± 1.98	7.58 ± 0.86

Values are presented as mean ± SD from seven human subjects. For each brain region, standard errors are listed in parentheses and are expressed as the percentage of the variable itself.

**Figure 5:**
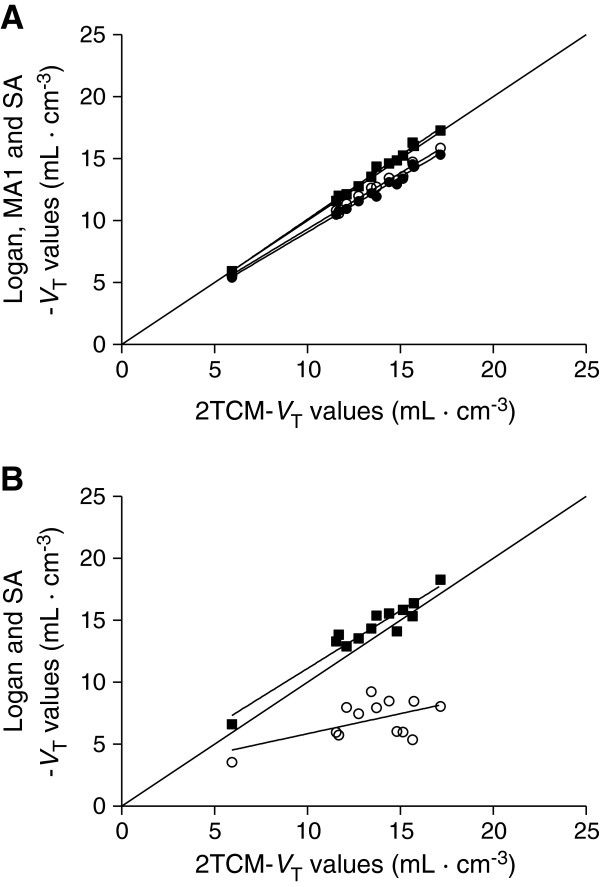
**Regression analyses for *V***_**T **_**results obtained from the 13 brain regions of a 23-year-old subject. (A)** Spectral analysis (filled square) provided good results with a very small bias (+1.4%). Logan (empty circle) and MA1 (filled circle) slightly underestimated *V*_T_ values (-7.4% and -9.7%, respectively, for this subject). At the voxel level **(B)**, only SA provided results with a small bias and good correlation with 2TCM (0.932*x* + 1.793, *R*^2^ = 0.92, *p* < 0.0001). In contrast, Logan_voxel_ underestimated *V*_T_ values by about 47% (0.324*x* + 2.61, *R*^2^ = 0.32, *p* = 0.047).

As regards the second point, Logan_voi_ and MA1_voi_ underestimated *V*_T_ values by about 10% (Figure 5A), and these differences were statistically significant (*p* < 0.05; factorial repeated measures ANOVA using Bonferroni adjustment, *F*_1.33,8.01_ = 57.93). In contrast, *V*_T_ values obtained with SA_voi_ had an average bias of only +1% (range -7% to +6%) and were not statistically different from the reference values obtained with 2TCM (*p* > 0.05). Logan_voi_, MA1_voi_, and SA_voi_ correlated well with the reference *V*_T_ values obtained with the 2TCM: *R*^2^ was >0.90 for all subjects, except for one subject in whom *R*^2^ for SA_voi_ was 0.73. Logan_voxel_ severely underestimated *V*_T_, with an average difference of -40% (*p* < 0.05) (Figure 5B). Moreover, linear regression analysis showed that Logan_voxel_ was significantly correlated to 2TCM *V*_T_ values in only four of seven subjects. The parametric images obtained with MA1 (MA1_voxel_) contained many noisy voxels that prevented reliable quantification. These noisy voxels were located predominantly, but not exclusively, in areas where blood flow (*K*_1_) is low, such as the white matter. Attempts to eliminate these noisy voxels by adjusting *K*_1_ values were not successful. Therefore, MA1_voxel_ was not considered for further analysis. Compared to 2TCM, *V*_T_ values obtained with SA_voxel_ showed a mean bias of only +2% (range, -10% to +7%) and were not statistically different (*p* > 0.05) from the reference values. Predictably, SA_voxel_ values were higher than Logan_voi,_ Logan_voxel_, and MA1_voi_ (*p* < 0.05). Finally, SA_voxel_ significantly correlated with 2TCM in all subjects: *R*^2^ for brain regions ranged from 0.81 to 0.95, except in one subject who had an *R*^2^ of 0.60.

### Use of a venous input function

Logan-*V*_T_ results obtained with a full venous input function were consistent with Logan-*V*_T_ results obtained with an arterial plasma input function in six subjects (with an error <10% compared to the reference values obtained with the arterial input function). However, in one subject, Logan-*V*_T_ was overestimated by 27%.

## Discussion

This study sought to quantify [^11^C]befloxatone binding in human brain using arterial blood samples and PET imaging. We also assessed whether arterial cannulation could be avoided by replacing arterial samples with arterialized venous samples.

We found that [^11^C]befloxatone binding is better quantified using a two-tissue compartment model or SA. Venous samples cannot, however, be a reliable substitute for arterial samples.

Despite a slow washout of the radioligand from the brain, *V*_T_ values were well identified with an unconstrained two-tissue compartment model (mean standard error, 4.7%). A constrained 2TCM did not provide a clear advantage for *V*_T_ estimation over the unconstrained model. *V*_T_ values were higher in the basal ganglia and the cortical structures (about 12 to 19 mL/cm^3^) and lower in the cerebellum (about 7 mL/cm^3^). These values were slightly lower than the *V*_T_ values previously found in baboons, where the *V*_T_ was 19 to 23 mL/cm^3^ for high-uptake regions and 10 to 11 mL/cm^3^ for the cerebellum [12]. Notably, in a previous study in baboons, we used a three-compartment model, corresponding to the free ligand in the tissue, the ligand specifically bound to the receptors and the nonspecific binding [14]. However, the concentration of the nonspecific binding was very low in all regions, and the equilibrium between the free and the nonspecific ligand was reached relatively quickly, compared with the equilibrium between the other compartments [14]. Therefore, the nonspecific and the free compartments of [^11^C]befloxatone can be lumped together in a two-tissue model.

Compared with 2TCM, both the Logan plot (Logan_voi_) and the multilinear analysis (MA1_voi_) significantly underestimated *V*_T_ values in large brain regions (about -10%). In contrast, the average bias of *V*_T_ values obtained with standard SA_voi_ was only 1%.

Owing to the sensitivity of the Logan plot to noise, especially at the voxel level, Logan_voxel_ severely underestimated *V*_T_ values (-40% on average) and did not significantly correlate with 2TCM *V*_T_ in three out of the seven subjects. Multilinear analysis has been shown to be more robust to noise-induced bias [20]. However, parametric images (MA1_voxel_) contained numerous noisy voxels and were therefore not amenable to quantification. SA was the only technique capable of providing good results at the voxel level. Indeed, SA_voxel_ showed only a small, nonsignificant bias (+2%) compared to *V*_T_ values obtained with 2TCM and correlated well with 2TCM for all subjects. As this study shows, when the most commonly used graphical approaches fail to provide parametric images of good quality, SA can be a valid alternative. The use of SA has long been hampered by the complexity of its implementation. However, new user-friendly software, such as SAKE or Pmod 3.4, may encourage its use for clinical protocols where statistical parametric mapping analyses are performed.

Autoradiography and immunohistochemistry data have demonstrated that MAO-A is widespread in the brain [27,28]. Moreover, studies of nonhuman primates found that [^11^C]befloxatone binding was displaceable in all brain regions [12]. These data suggest that a reference region may not exist to quantify [^11^C]befloxatone binding. Without a reference region, quantification can only be achieved by using an arterial plasma input function, which is an invasive procedure. In addition, measuring the arterial input function usually requires labor-intensive HPLC analyses. However, one of the favorable characteristics of [^11^C]befloxatone is that no radiometabolites are detected in the plasma. In consequence, [^11^C]befloxatone input function can be easily achieved using only plasma isolation without HPLC analyses. Notably, our previous analysis in baboons showed that [^11^C]befloxatone radiometabolites constituted about 20% of plasma radioactivity at 30 min after injection [14]. This discrepancy was due to interspecies differences and possibly linked to the faster metabolism of baboons.

To avoid arterial samples altogether, we sought to validate the use of an input function composed of arterialized venous samples. Indeed, arterialized venous samples are routinely used in several institutions as a replacement for arterial samples. A stable arteriovenous equilibrium began from about 3 min after injection; therefore, the early portions of the arterial and venous input functions (i.e., the peaks) were dissimilar. This is a common finding with most tracers and molecules and is linked to the diffusion of the radiotracer in its volume of distribution [29,30]. A different peak shape would prevent the use of a venous full input in association with compartmental modeling or SA. In contrast, the Logan plot does not require that the shape of the peak be perfectly estimated because it relies on the total area under the curve of the input function. Indeed, kinetic modeling performed using a venous input function yielded Logan-*V*_T_ values which were consistent with those obtained from arterial sampling: six out of seven subjects had Logan-*V*_T_ estimation errors smaller than 10%. There was, however, one major outlier with an error of 27%, and this suggests that arterialized samples might not be a reliable alternative to the full arterial input function for [^11^C]befloxatone imaging. Notably, this subject was the only one to have a significant deviation from arteriovenous equilibrium at a late time. However, the degree of arterialization of the venous blood for this subject (vein/artery ratio of 0.74 for pO_2_ and 1.03 for pCO_2_) was similar to that of the others (0.77 ± 0.08 and 1.01 ± 0.01 for pO_2_ and pCO_2_, respectively). It is possible that a higher degree of arterialization could have been achieved using a different warming technique [31]. It should, however, be noted that, even with optimal conditions, arterialization of venous blood is difficult to achieve and is plagued by intersubject variability [32]. Therefore, unless a better equilibrium can be achieved with a different technique, an arterial input function will be necessary for the quantification of [^11^C]befloxatone binding.

Finally, it should be noted that despite having several good imaging characteristics (high specific binding, low metabolism in plasma), [^11^C]befloxatone has one important drawback, i.e., a slow washout from the brain. Although *V*_T_ values were well identified, it would nevertheless be preferable to have a radioligand with a faster washout, even if there was a higher rate of metabolism in the plasma (provided that radiometabolites do not enter into the brain).

## Conclusions

Binding of [^11^C]befloxatone to MAO-A can be quantified using an arterial input function and a two-compartment model or, in parametric images, with spectral analysis.

## Competing interests

The authors declare that they have no competing interests.

## Authors' contributions

PZF conceived of the study, performed the image analyses and statistical comparisons, and drafted the manuscript. CL, DR, CT, JLM, and MB recruited the subjects, acquired the image and blood data and participated in the data analysis and the design, coordination, and writing of the manuscript. All authors read and approved the final manuscript.
